# Supplementary material for: HIV prevalence in transgender women and cisgender men who have sex with men in sub-Saharan Africa
Source: AIDS. 2026 Feb 12;40(4):510–6. doi: 10.1097/QAD.0000000000004405 (PMC12955963; doi:10.1097/QAD.0000000000004405)
Supplement: Supplementary file 1 [file aids-40-510-s001.docx]

**HIV prevalence in transgender women and cisgender men who have sex with men in sub-Saharan Africa 2010-2022: a meta-analysis** - Supplementary information

**Suplementary Table S1:** Studies included in meta-analysis of HIV prevalence in transgender women (TGW) and cis-gendered men who have sex with men (cis-MSM)

| Study | Year of publication | Country | Year of data collection | Recruitment method | Transgender women inclusion criterion | TGW prevalence  (%; n/N) | cis-MSM prevalence (%; n/N) |
| --- | --- | --- | --- | --- | --- | --- | --- |
| Stahlman et al. ^15^ | 2016 | Burkina Faso | 2013 | RDS | Identified as female or transgender, assigned male sex at birth, and reported having had insertive or receptive anal sex with a man in the past 12 months | 0.0% (0/5) | 4.5% (1/22) |
|  |  | Côte d’Ivoire | 2015 |  |  | 23.5% (16/68) | 7.3% (4/55) |
|  |  | Togo | 2013 |  |  | 22.2% (2/9) | 7.1% (3/42) |
| Keshinro et al. ^24^ | 2016 | Nigeria | 2016 | RDS | Identified as female, assigned male sex at birth, and reported having had insertive or receptive anal sex with a man in the past 12 months | 51.5% (123/239) | 40.7% (786/1929) |
| Botswana MoH ^26^ | 2017 | Botswana | 2017 | RDS | Identified as a woman, formerly or currently biologically male | 50.0% (6/12) | 14.9% (110/736) |
| Ghana MoH ^59^ | 2017 | Ghana | 2017 | RDS | Identified as a woman, biologically male, and have had anal or oral sex with another man in the last 12 months | 28.1% (9/31) | 17.4% (650/3723) |
| Mozambique MoH ^49^ | 2018 | Mozambique | 2017 | PLACE | Identified as a woman or transgender, assigned male at birth | 12.7% (15/118) | 9.9% (51/517) |
| Poteat et al. ^12^ | 2017 | Burkina Faso | 2013 | RDS | Self-identified as transgender or female/woman, and assigned male sex at birth | 2.8% (3/108) | 3.3% (18/552) |
|  |  | Côte d’Ivoire | 2016 |  |  | 25.5% (76/298) | 8.0% (61/766) |
|  |  | Gambia | 2011 |  |  | 50.0% (2/4) | 9.0% (18/200) |
|  |  | Lesotho | 2014 |  |  | 59.2% (42/71) | 28.6% (130/455) |
|  |  | Malawi | 2012 |  |  | 16.0% (12/75) | 15.2% (40/263) |
|  |  | Senegal | 2015 |  |  | 37.2% (74/199) | 27.8% (147/528) |
|  |  | Eswatini | 2011 |  |  | 14.2% (17/120) | 18.6% (38/204) |
|  |  | Togo | 2013 |  |  | 17.6% (9/51) | 8.5% (53/626) |
| Angola MoH ^51^ | 2018 | Angola | 2017 | PLACE | Identified as a woman, and assigned male at birth | 9.0% (8/89) | 2.0% (20/1016) |
| Hakim et al. ^50^ | 2018 | Mali | 2015 | RDS | Identified as a woman, assigned male sex at birth, and reported having had insertive or receptive anal sex with a man in the past 6 months | 24.3% (9/37) | 9.7% (12/124) |
| Jobson et al. ^57^ | 2018 | South Africa | 2017 | Snowball | Self-identified as female or transgender, biologically male, and as having sex with men | 57.1% (12/21) | 31.3% (47/150) |
| Sandfort et al. ^58^ | 2019 | Kenya | 2016 | Convenience | Did not identify as male, assigned male sex at birth, and ever had sex with a man | 25.0% (8/32) | 14.6% (20/137) |
|  |  | Malawi | 2016 |  |  | 31.6% (12/38) | 17.9% (15/84) |
|  |  | South Africa | 2016 |  |  | 57.6% (38/66) | 36.9% (90/244) |
| Liberia MoH ^61^ | 2019 | Liberia | 2018 | RDS | No inclusion criterion provided | 27.6% (80/290) | 37.9% (240/633) |
| Fearon et al. ^13^ | 2020 | South Africa | 2017 | RDS | Assigned male at birth and ﻿current identity was female or transgender. Sex with a man in last 12 months | 44.6% (20/44) | 38.6% (90/233) |
| Malawi MoH ^25^ | 2020 | Malawi | 2020 | RDS | Self-identified as female or transgender. Engaged in sexual activities with other men in the past 12 months | 13.5% (73/540) | 12.5% (178/1425) |
| Nigeria MoH ^47^ | 2020 | Nigeria | 2020 | Venue-based | Identifies him/herself as a transsexual and undertakes sexual activity with a man | 18.5% (775/4190) | 21.1% (926/4397) |
| Rwema et al. ^52^ | 2020 | Rwanda | 2018 | RDS | Self-identified as transgender or a woman, assigned male sex at birth, and reported having had insertive or receptive anal sex with a man in the past 12 months | 9.4% (10/106) | 10.2% (64/630) |
| eSwatini MoH ^46^ | 2021 | Eswatini | 2020 | RDS | Identified as female. Report have insertive or receptive anal sex with a man in the past 12 months | 41.2% (7/17) | 20.2% (84/415) |
| Smith et al. ^53^ | 2021 | Kenya | 2017 | RDS | Male sex assignment at birth, self-identify as female or transgender, and having had consensual anal or oral sexual activity with a man in the previous 12 months | 40.0% (28/70) | 28.9% (151/522) |
| Sullivan et al. ^54^ | 2021 | South Africa | 2016 | Convenience | Male sex at birth and current gender identity as female or transgender, and report having had insertive or receptive anal sex with a man in the past 12 months | 59.1% (13/22) | 41.8% (110/263) |
| Harris et al. ^55^ | 2022 | Zimbabwe | 2019 | RDS | Born biologically male; self-identifed as a woman, transgender, or genderqueer, and engaged in anal or oral sex with a man in the past 12 months | 27.5% (92/335) | 21.1% (248/1176) |
| Sierra Leone MoH ^56^ | 2022 | Sierra Leone | 2021 | RDS | A woman who had a gender identity that is different from her sex at birth | 4.7% (23/487) | 4.2% (18/430) |
| Zambia MoH ^62^ | 2023 | Zambia | 2021 | RDS | Self-identified as a woman, biologically male at birth and self-reported anal or oral sex with biological male in past 6 months | 17% (77/453) | 13.5% (147/1088) |

RDS=respondent driven sampling ^36^; PLACE=Priorities for Local AIDS Control Efforts ^37^

| **Study** | **Area** | **Year** | **HIV prevalence (%)** | | | | |
| --- | --- | --- | --- | --- | --- | --- | --- |
|  |  |  | **TGM** | **TGW** | **cis-MSM** | **15-49 women** | **15-49 men** |
| Benin MoH ^36^ | Benin | 2020 | **9.5** | **26.4** | *7* ^49^ | *1.1* | *0.5* |
| Mujugira et al. ^42^ | Kampala, Uganda | 2020 | **4.1** | *20.0* ^17^ | *12.7* ^63^ | *7.4* | *4.8* |
| Botswana MoH ^27^ | Botswana | 2017 | **6.8** | **45.5** | **14.9** | *26.2* | *16* |
| Malawi MoH ^43^ | Malawi | 2021 | **23.9** | **62.3** | *12.5* ^26^ | *9.3* | *5.6* |
| Sierra Leone ^40^ | Sierra Leone | 2021 | **1.11** | **4.36** | **3.4** | *1.8* | *1* |

**Supplementary Table S2:** Estimates of HIV prevalence among transgender men (TGM) compared with transgender women (TGW), cis-gendered men who have sex with men (cis-MSM), and 15-49 total population. In each row, estimates in bold are from the same study indicated in the ‘Study’ column. Transgender women and cis-MSM estimates in italics are the best available comparator estimates from other studies. Total population HIV prevalence estimates are from UNAIDS estimates derived using Spectrum and Naomi models.
